# Development and validation of a novel qualitative test for plasma fibrinogen utilizing clot waveform analysis

**DOI:** 10.1038/s41598-021-04464-5

**Published:** 2022-01-21

**Authors:** Atsuo Suzuki, Nobuaki Suzuki, Takeshi Kanematsu, Sho Shinohara, Hiroshi Kurono, Nobuo Arai, Shuichi Okamoto, Naruko Suzuki, Shogo Tamura, Ryosuke Kikuchi, Akira Katsumi, Tetsuhito Kojima, Tadashi Matsushita

**Affiliations:** 1 Department of Medical Technique, Nagoya University Hospital, 65 Tsurumai-cho, Showa-ku, Nagoya, 466-8560 Japan; 2 Department of Transfusion Medicine, Nagoya University Hospital, Nagoya, Aichi Japan; 3 Department of Clinical Laboratory, Nagoya University Hospital, Nagoya, Aichi Japan; 4 Sysmex Corporation, Kobe, Hyogo Japan; 5 Department of Hematology-Oncology, Nagoya University Graduate School of Medicine, Nagoya, Aichi Japan; 6 Division of Cellular and Genetic Sciences, Department of Integrated Health Sciences, Nagoya University Graduate School of Medicine, Nagoya, Aichi Japan; 7 Department of Hematology, National Centre for Geriatrics and Gerontology, Obu, Aichi Japan; 8 Aichi Health Promotion Foundation, Nagoya, Aichi Japan

**Keywords:** Laboratory techniques and procedures, Haematological diseases

## Abstract

Plasma fibrinogen is commonly examined by Clauss fibrinogen assay, which cannot distinguish between quantitative and qualitative fibrinogen anomalies. However, our previously reported Clauss fibrinogen assay utilizing clot waveform analysis (Clauss-CWA) provides additional information that contributes to the classification of fibrinogen anomalies. In this study, we adopted the Clauss-CWA method for an autoanalyzer to automatically measure the antigenic estimate (eAg) of fibrinogen in addition to the functional amount (Ac), and to thus provide the Ac/eAg ratio as a qualitative indicator. Performance was validated by receiver operating characteristics (ROC) and precision recall (PR) curve analyses using a patient cohort, consisting of a training cohort (n = 519) and a validation cohort (n = 523), both of which contained cases of congenital (hypo)dysfibrinogenemia as qualitative defects. We obtained an optimal cutoff of 0.65 for Ac/eAg by ROC curve analysis of the training cohort, offering superior sensitivity (> 0.9661) and specificity (1.000). This cutoff was validated in the validation cohort, providing positive predictive value > 0.933 and negative predictive value > 0.998. PR curve analysis also showed that Clauss-CWA provided excellent performance for detecting qualitative fibrinogen anomalies. The Clauss-CWA method may represent a useful approach for detecting qualitative fibrinogen abnormalities in routine laboratory testing.

## Introduction

Plasma fibrinogen can be examined by several assays. In routine laboratory testing, thrombin time and the Clauss fibrinogen assay have each been recommended as an initial screening test^1,2^. The Clauss fibrinogen assay is well optimized for a large population of automated coagulation analyzers and is in wide use, and allows determination of functional fibrinogen levels.

We sometimes encounter low levels of plasma fibrinogen and suspect fibrinogen abnormalities from routine screening tests. The majority of cases of congenital dysfibrinogenemia and hypodysfibrinogenemia are diagnosed incidentally^3^, and most patients diagnosed with congenital dysfibrinogenemia actual appear asymptomatic^3–8^. Such individuals show significantly low levels of functional fibrinogen, but discriminating between low levels of functional fibrinogen due to qualitative defects and low levels of fibrinogen antigen due to quantitative defects by Clauss fibrinogen assay alone is quite difficult. Hence, for the laboratory diagnosis of fibrinogen abnormalities, antigenic measurement of fibrinogen is required^2^. However, measurement of fibrinogen antigen incurs extra costs over functional fibrinogen testing and is unsuitable for routine testing due to its low throughput. Furthermore, the precise prevalence of qualitative fibrinogen deficiencies has not been established because of the large number of asymptomatic cases^9^, representing another reason antigenic fibrinogen determination is not carried out in every laboratory.

We recently reported that clot waveform analysis (CWA) in the Clauss fibrinogen assay could be useful to detect functional fibrinogen abnormalities with no additional measurement of fibrinogen antigen^10^. This method of Clauss fibrinogen assay utilizing CWA (Clauss-CWA) employs a value of maximum velocity (commonly represented as “Min1”^11^) and calculates the estimated fibrinogen antigen (eAg) levels introduced from the Min1 value. This eAg can be used as an alternative for actually measured fibrinogen antigen determined by immunological assays, and the ratio of functional fibrinogen level (Ac) to eAg (Ac/eAg ratio) can be used to identify qualitative fibrinogen defects. The clinical utility of this novel analytical method requires validation^12^.

The original method requires the export of data from an autoanalyzer and external calculation of the Ac/eAg ratio using spreadsheets. We therefore developed and installed novel autoanalysis software in a CN-6000 automated blood coagulation analyzer (Sysmex, Kobe, Japan). This software, which is still under development, immediately provides the Ac/eAg ratio from internal auto-calculations performed with each measurement. The present study aimed to validate this novel application and assess its usefulness in clinical settings using a larger cohort.

## Results

### Patient demographics

**Figure 1:**
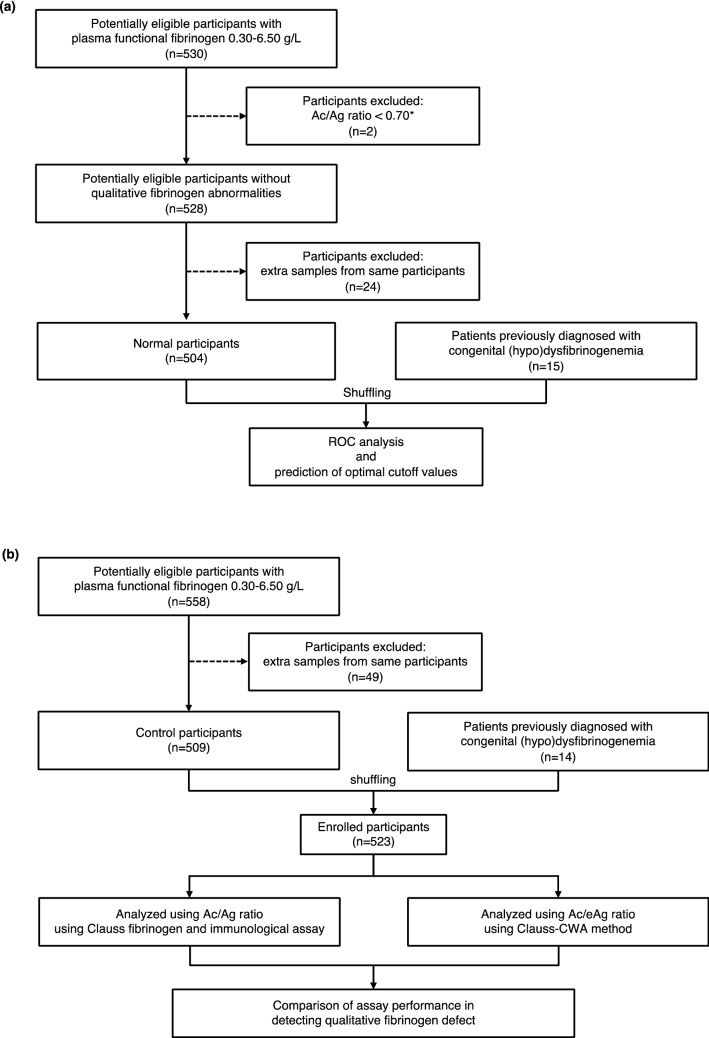
Study flow diagram. Study flow diagrams for the training cohort (**a**) and validation cohort (**b**). The cutoff value (*) to exclude participants showing lower Ac/Ag ratios was used according to the previous suggestion^30^. ROC, receiver operating characteristic; CWA, clot waveform analysis.

During the study period, plasma samples from 1044 patients were used, distributed across training and validation cohorts without any sample overlap between the two cohorts. The study flow diagram describing the patients and samples used in the training and validation cohorts is shown in Fig. 1.

From June 2020 to July 2020, a total of 530 samples were randomly collected and investigated for fibrinogen Ac and Ag. Two participants (samples) were excluded due to low Ac/Ag ratios in the absence of a previously confirmed diagnosis of congenital (hypo)dysfibrinogenemia (CD). After excluding overlapping participants, 504 samples were enrolled as a normal group representing normal Ac/Ag ratios, and a final total of 519 participants were enrolled after including 15 samples from 15 patients with CD in the training cohort. In addition, from August 2020 to October 2020, a total of 558 plasma samples were randomly collected. After excluding extra samples from the same individuals, 509 samples were enrolled as a control group. Plasma samples from 14 patients with CD diagnoses were also included and randomly assorted among the samples. In total, 523 participants were included in this validation cohort.

Participants and sample characteristics for the two study cohorts are provided in Table 1. Median age for the training cohort was 65 years (range 0–93 years), and 42.9% were female. Median fibrinogen Ac was 2.79 using Thrombocheck FibL (TCFibL) and 2.72 g/L using Dade Thrombin reagent (Dade), and Ag was 2.53 g/L. In the validation cohort, 509 samples were shuffled with 14 plasma samples from CD patients, and a final total of 523 participants were enrolled. Median age for the validation cohort was 62 (range 1–101), and 50.1% were female. Median fibrinogen Ac was 2.94 (TCFibL) and 2.82 g/L (Dade), and Ag was 2.64 g/L. No significant differences in age or sex were observed between the training and validation cohorts.

**Table 1:** Participants and sample characteristics of the study cohorts.

Characteristics	Training cohort	Validation cohort	*P* value*
**Participants**			
Total (n)	519	523	
Congenital (hypo)dysfibrinogenemia (n)	15	14	
Age (years)	65 (0–93)	62 (1–101)	0.7716
Female sex	223 (42.9)	262 (50.1)	0.0217
**Coagulation tests**			
PT (s)	11.3 (9.8–77.7) [1]	11.0 (9.5–29.3) [2]	< 0.0001
PT-INR	1.04 (0.89–8.68) [1]	1.01 (0.87–2.85) [2]	< 0.0001
APTT (s)	30.9 (21–200<) [1]	30.1 (21.9–84.7) [2]	< 0.0001
**Chemistry tests**			
AST (U/L)	22 (10–354) [9]	21 (8–258) [16]	0.0085
ALT (U/L)	18 (4–824) [9]	16 (3–221) [16]	0.0428
Total bilirubin (mg/dL)	0.7 (0.1–27.9) [10]	0.7 (0.2–28.4) [17]	0.1084
**Fibrinogen Ac (g/L)**			
Thrombocheck Fib(L)	2.79 (0.17–7.25) [1†]	2.94 (0.13–6.70)	0.0038
Dade^®^ Thrombin Reagent	2.72 (0.30–7.01)	2.82 (0.19–6.45)	0.0046
Fibrinogen Ag (g/L)	2.53 (0.64–6.99)	2.64 (0.78–7.10)	0.0052
**Ac/Ag ratio**			
Thrombocheck Fib(L)	1.07 (0.08–1.62) [1]	1.07 (0.06–1.43)	0.1400
Dade^®^ Thrombin Reagent	1.03 (0.15–1.32)	1.04 (0.07–1.30)	0.1007
**Fibrinogen eAg (g/L)**			
Thrombocheck Fib(L)	3.08 (0.48–8.41) [1]	3.27 (0.50–7.73)	0.0013
Dade^®^ Thrombin Reagent	2.95 (0.49–8.39)	3.17 (0.53–7.41)	0.0006
**Ac/eAg ratio**			
Thrombocheck Fib(L)	0.90 (0.30–1.25) [1]	0.89 (0.17–1.10)	0.0017
Dade^®^ Thrombin Reagent	0.90 (0.29–0.92)	0.89 (0.22–1.010	0.0001

Continuous valuables are reported as median (range), categorical variables are reported as n (%), and numbers of missing data, if any, are listed inside brackets ([N]).

*Based on Mann–Whitney test for continuous variables and Fisher's exact test for categorical variables.

^†^No coagulation in Clauss fibrinogen assay.

### Analytical precision

We first investigated the analytical precision of our newly developed fibrinogen analysis utilizing CWA. When TCFibL was used, the precision of fibrinogen Ac measurement was 2.6% (control plasma N [CPN]) or 3.9% (control plasma P [CPP]), whereas that of eAg was 2.1% (CPN) or 4.7% (CPP). Using Dade, the precision of Ac was 2.6% (CPN) or 4.0% (CPP), and that of eAg was 2.5% (CPN) or 2.6% (CPP). On the other hand, the precision of fibrinogen Ag determination was 3.1% for normal levels and 3.7% for low levels.

### Training of Ac/eAg ratio

Ac/eAg ratio was evaluated as an alternative index to distinguish qualitative from quantitative abnormalities of plasma fibrinogen. The results of receiver operating characteristics (ROC) analyses are shown in Table 2. When TCFibL was used, the area under the ROC curve (AUROC) of the Ac/eAg ratio for distinguishing CD from controls was 0.9661 (95% confidence interval [CI] 0.9018–1.000), and the optimal cutoff value was predicted as 0.62–0.66. Sensitivity and specificity were 0.9286 (95% CI 0.6853–0.9963) and 1.000 (95% CI 0.9925–1.000), respectively. The positive likelihood ratio (+LR) was infinity and the negative likelihood ratio (−LR) was 0.071 (95% CI 0.01–0.5). When Dade was used, AUROC was 0.9962 (95% CI 0.9890–1.000) and the predicted cutoff of Ac/eAg ratio was 0.60–0.68. Sensitivity, specificity, + LR, and − LR were 0.9333 (95% CI 0.7018–0.9960), 1.000 (95% CI 0.9925–1.000), infinity, and 0.067 (95% CI 0.01–0.4), respectively.

**Table 2:** Predictive cutoff values and their performance in distinguishing qualitative fibrinogen defects.

	TCFibL	Dade
Predictive cutoff for Ac/eAg ratio	0.62–0.66	0.60–0.68
AUROC (95% CI)	0.9661 (0.9018–1.000)	0.9962 (0.9890–1.000)
Sensitivity (95% CI)	0.9286 (0.6853–0.9963)	0.9333 (0.7018–0.9960)
Specificity (95% CI)	1.000 (0.9925–1.000)	1.000 (0.9925–1.000)
+ LR (95% CI)	Infinity	Infinity
− LR (95% CI)	0.071 (0.01–0.5)	0.067 (0.01–0.4)

TCFibL, Thrombocheck FibL reagent; Dade, Dade Thrombin Reagent; AUROC, area under the receiver-operating characteristics curve; LR, likelihood ratio; + LR, positive LR; − LR, negative LR; Ac, functional fibrinogen concentration; Ag, antigenic fibrinogen concentration; eAg, estimated Ag.

### Validation of the predictive performance of Ac/eAg ratio

**Figure 2:**
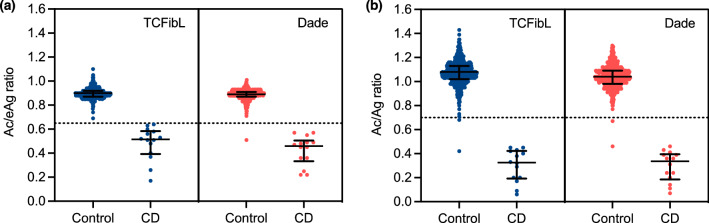
Comparison of Ac/eAg and Ac/Ag ratio in validation cohort. A scatter plot for Ac/eAg ratio (**a**) and Ac/Ag ratio (**b**) analyzed in the validation cohort. The left area shows results obtained with Thrombocheck FibL reagent (TCFibL, blue), and the right area shows results obtained with Dade Thrombin Reagent (Dade, rose). The dotted line shows the predicted cutoff of 0.65 (**a**) or the previously suggested cutoff of 0.70 (**b**). Bars show median with interquartile range. CD, congenital (hypo)dysfibrinogenemia.

Optimal cutoffs revealed from the training cohort were used to evaluate the predictive performance of Ac/eAg ratio. We used the suggested cutoff of 0.65 for both reagents and found that CD could be clearly distinguished with high sensitivity and specificity (Fig. 2A). When TCFibL was used, sensitivity and specificity were 1.000 (95% CI 0.7847–1.000) and 1.000 (95% CI 0.9925–1.000), respectively, and positive predictive value (PPV) and negative predictive value (NPV) were both 1.00 (Table 3). When using Dade, sensitivity, specificity, PPV, and NPV were 1.000 (95% CI 0.7847–1.000), 0.9961 (95% CI 0.9858–0.9993), 0.933, and 0.988, respectively.

**Table 3:** Predictive accuracies of Ac/Ag ratio and Ac/eAg ratio for qualitative fibrinogen defect in validation cohort.

Index	Reagent	AUROC	Cutoff	Sensitivity (95% CI)	Specificity (95% CI)	PPV	NPV
Ac/eAg ratio	TCFibL	1.000 (1.000–1.000)	0.65	1.000 (0.7847–1.000)	1.000 (0.9925–1.000)	1.000	1.000
	Dade	0.9996 (0.9986–1.000)	0.65	1.000 (0.7847–1.000)	0.9961 (0.9858–0.9993)	0.933	0.998
Ac/Ag ratio	TCFibL/FAFbg	0.9995 (0.9984–1.000)	0.70^¶^	1.000 (0.7847–1.000)	0.9941 (0.9828–0.9984)	0.875	0.996
			0.55^§^	1.000 (0.7847–1.000)	0.9980 (0.9890–0.9999)	0.933	0.998
	Dade/FAFbg	0.9999 (0.9996–1.000)	0.70^¶^	1.000 (0.7847–1.000)	0.9961 (0.9858–0.9993)	0.875	0.996
			0.55^§^	1.000 (0.7847–1.000)	0.9980 (0.9890–0.9999)	0.933	0.998

AUROC, area under the receiver-operating characteristics curve; CI, confidence interval; PPV, positive predictive value; NPV, negative predictive value; Ac, functional fibrinogen concentration; Ag, antigenic fibrinogen concentration; eAg, estimated Ag; TCFibL, Thrombocheck FibL reagent; Dade, Dade Thrombin Reagent; FAFbg, FactorAuto Fibrinogen.

^¶^Cutoff value suggested by Krammer et al^30^.

^§^Cutoff value proposed in the current study.

We also validated the cutoff of 0.70 for Ac/Ag ratio by ROC analysis and found that sensitivity and specificity were 1.000 (95% CI 0.7847–1.000) and 0.9941 (95% CI 0.9828–0.9984) for TCFibL, and were 1.000 (95% CI 0.7847–1.000) and 0.9961 (95% CI 0.9858–0.9993) for Dade, respectively. PPV was 0.875 and NPV was 0.996 for both TCFibL and Dade in combination with FactorAuto Fibrinogen reagent (FAFbg). On the other hand, the cutoff of 0.70 for Ac/Ag ratio was generally used for the screening of qualitative fibrinogen abnormalities, even though proper validation has not been performed^12^. We therefore performed ROC analysis of the validation cohort to reveal an optimal cutoff Ac/Ag ratio, yielding an optimal cutoff of 0.55 for Ac/Ag ratio. This cutoff of 0.55 yielded sensitivity of 1.000 (95% CI 0.7847–1.000) and specificity of 0.9980 (95% CI 0.9890–0.9999) for both reagents. PPV and NPV were 0.933 and 0.998, respectively, when either reagent was used.

### Precision–recall curve analysis

**Figure 3:**
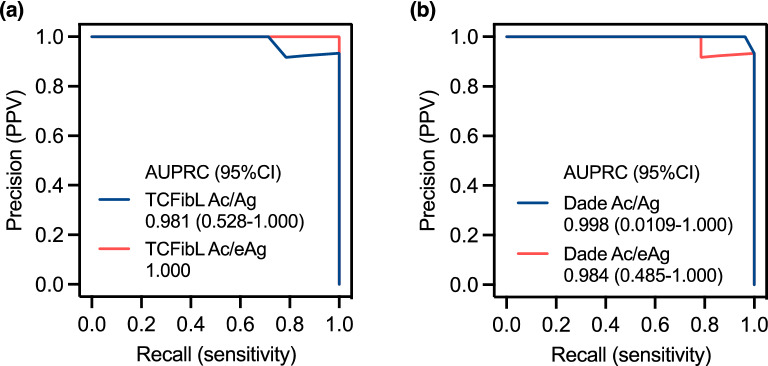
Precision–recall curve analysis. Precision–recall (PR) curves in TCFibL (**a**) and Dade (**b**) reagents. The X-axis represents the recall (sensitivity) and the Y-axis represents precision (positive predictive value [PPV]). The blue solid line shows the results for Ac/Ag ratio and the rose-colored line shows the results for Ac/eAg ratio. The number represents the area under the PR curve (AUPRC) with 95% confidence interval (CI).

While generally ROC analysis has been used to evaluate the diagnostic performance of laboratory tests or biomarkers, the precision recall (PR) curve analysis would be useful to estimate performance in cases of low-prevalence diseases^13^ or to analyze imbalanced datasets^14^*.* We compared the PR curve and area under the PR curve (AUPRC) between Ac/eAg ratio and Ac/Ag ratio. Figure 3A shows the PR curves for Ac/Ag ratio and Ac/eAg ratio in the validation cohort when TCFibL was used. Both showed excellent PR curves, and AUPRC was 0.981 (95% CI 0.528–1.000) for Ac/Ag and 1.000 (95% CI 1.000–1.000) for Ac/eAg. PR curves when Dade was used are shown in Fig. 3B, and were also good. AUPRC was 0.998 (95% CI 0.0109–1.000) for Ac/Ag and 0.984 (95% CI 0.485–1.000) for Ac/eAg ratio.

### Outliers

As shown in Fig. 2, the two samples (from the two different individuals) were recognized as outliers in the control group showing a lower Ac/(e)Ag ratio than the predicted cutoffs. The two different individuals, here termed Patients C1 and C2, became the outliers in the validation cohort, and one represented a discordant result when the different reagent was used.

Patient C1 was an 80-year-old man with chronic disseminated intravascular coagulation (DIC) due to thoracic aortic aneurysm (TAA). He showed high levels of D-dimer (75.0 µg/mL), thrombin-antithrombin complex (TAT) (56.0 ng/mL), and plasmin-α2-plasmin inhibitor complex (PIC) (19.32 µg/mL), indicating a hypercoagulable, hyperfibrinolytic state. He showed reduced fibrinogen Ac (0.49 by TCFibL, 0.54 g/L by Dade) and moderately reduced Ag (1.16 g/L), and consequently displayed a low Ac/Ag ratio (0.42 by TCFibL, 0.46 by Dade). When Dade was used, fibrinogen eAg and Ac/eAg ratio were 1.06 g/L and 0.51, respectively, compatible with the Ac/Ag ratio. When TCFibL was used, fibrinogen eAg was 0.70 g/L and Ac/eAg ratio was 0.69, higher than the predicted cutoff and thus showing discordant results between different reagents.

Patient C2 was a 76-year-old man also diagnosed with chronic DIC due to TAA. Plasma D-dimer was 62.3 µg/mL, fibrin/fibrinogen-degradation products (FDPs) were 190.3 µg/mL, TAT was 51.5 ng/mL, and PIC was 12.12 µg/mL. Fibrinogen Ac was 1.23 with TCFibL and 1.22 g/L with Dade, and Ac/eAg ratios were 0.79 (TCFibL) and 0.73 (Dade), within normal range. Fibrinogen Ag was 1.82 g/L, and Ac/Ag ratio was 0.68 with TCFibL and 0.67 with Dade, slightly lower than the suggested cutoff. A discordant result was seen between Ac/Ag and Ac/eAg ratio used for categorization, but on the other hand, when the cutoff of 0.55 was used, Patient C2 was not categorized as an outlier and was concordant with the results for the Ac/eAg ratio.

## Discussion

Presence of a discrepancy between functional and immunoreactive fibrinogen is an essential indicator in diagnosing qualitative disorders of fibrinogen. The standard method to identify such discrepancies is to perform both Clauss fibrinogen assay (thrombin time method) and immunological assay. Both previous^15^ and recent^16^ studies have clarified the utility of prothrombin time (PT)-derived fibrinogen assay for discriminating qualitative fibrinogen defects from quantitative defects in combination with the Clauss fibrinogen assay. The present study established an automated analysis that enables evaluation of the quality and quantity of plasma fibrinogen using Clauss-CWA method. In response to comments in the review article by Casini^12^, we validated automated analysis software using clinical samples and found that Clauss-CWA was useful to distinguish qualitative and quantitative abnormalities in routine testing. These results indicated that the Clauss fibrinogen assay commonly used for routine laboratory testing could perform both quantitative and qualitative analyses of plasma fibrinogen by adding CWA.

The automated Clauss-CWA showed excellent precision, sufficient for use in clinical settings. The diagnostic performance as evaluated by ROC and PR curve analyses was not inferior to the standard method using Ac/Ag ratio, and predictive cutoffs were almost equal between the two thrombin reagents. Our suggested cutoff for Ac/eAg ratio was 0.65 for both TCFibL and Dade reagents and clearly distinguished qualitative fibrinogen abnormalities, but the cutoff of 0.65 was lower than the suggested cutoff for Ac/Ag ration of 0.70. The differences in cutoff between Ac/eAg ratio and Ac/Ag ratio might be due to the calibrator used, which did not have an established value for “estimated” Ag. Indeed, the population of Ac/eAg ratio values differed somewhat from that of Ac/Ag ratio and median Ac/eAg ratio was slightly lower than median Ac/Ag ratio. Further, Ac/eAg ratios were not completely interchangeable with Ac/Ag ratios, nor were cutoff values. Reasons for the clear differences between Ag and eAg were not able to be elucidated, but we considered this would most likely be based on Michaelis–Menten kinetics. The conversion of fibrinogen to fibrin by thrombin shows Michaelis–Menten behavior^17^, and calibration curves for eAg resemble a Michaelis–Menten plot. The logarithmic relationship between Min1 and eAg becomes stronger at higher reaction velocities (= Min1). Consequently, eAg is actually calculated at high concentrations when plasma fibrinogen exceeds 3.0 g/L. The actual relationship between Ac and eAg shows a linear regression equation of y = 1.285x − 0.241 for TCFibL and y = 1.269x − 0.289 for Dade (Supplementary Fig. S1). These significant differences between Ag and eAg resulted in a median Ac/eAg ratio lower than the median Ac/Ag ratio. Although regression equations may be used to reach agreement between Ag and eAg, we considered such adjustment as unnecessary, since additional determination of Ag is needed.

On the other hand, although the suggested Ac/Ag ratio cutoff of 0.70 had not been prospectively validated^18^, the validation cohort used in the present study showed that the cutoff of 0.70 was acceptable. We introduced an optimal cutoff of 0.55 from ROC analysis, and the predictive performance of a cutoff of 0.55 was slightly better than that of 0.70, but further studies may need to reconsider optimal cutoffs for both Ac/Ag and Ac/eAg ratios.

The major cause of qualitative fibrinogen abnormalities would be congenital (hypo)dysfibrinogenemia (currently termed type 3 and 4 congenital fibrinogen disorders^2^), but the precise prevalence of congenital dysfibrinogenemia remains unclear^9^. One reason is that patients with inherited dysfibrinogenemia are frequently asymptomatic^9^, and 55% of more than 260 dysfibrinogenemia cases showed no clinical complications^4^. A recent study also revealed that more than half of patients with dysfibrinogenemia had been asymptomatic^6^. The current study used randomly collected clinical samples in the training cohort, and identified two samples (individuals) with suspected qualitative fibrinogen abnormalities that were excluded due to a reduced Ac/Ag ratio (< 0.70). The two individuals showed reduced functional fibrinogen (< 1.50 g/L) at the time of sample collection, but had previously had normal levels (data not shown), and also had no family history of congenital dysfibrinogenemia. The significant discrepancies between functional and antigenic fibrinogen seemed to be acquired in those two cases. Acquired (hypo)dysfibrinogenemia requires discrimination from acquired hypofibrinogenemia, and has been found infrequently in various diseases^19–23^. Actually, one of the two patients showed severe liver dysfunction with jaundice. The other patient suffered from severe DIC showing high levels of FDP (> 300 µg/mL), as did the outliers found in the validation cohort (Patients C1 and C2). As FDPs are known to competitively inhibit thrombin^24^ and prolong thrombin times at high levels^25^, we considered that the reduced Ac/Ag ratios were not attributable to acquired dysfibrinogenemia. Interestingly, Ac/eAg ratios as determined by TCFibL reagent were higher than the predicted cutoffs representing the normal quality of fibrinogen, and indicate that Clauss-CWA would not be less influenced by FDPs when evaluating fibrinogen quality.

Our data suggested that Clauss-CWA would be applicable for use in routine laboratory testing, but we recognize some limitations in the present study and the established method. First, we only included CD as a target disease, so no other acquired qualitative abnormalities were included or validated. Such acquired abnormalities are rare and thus difficult to enroll in study cohorts, so further prospective studies are needed to evaluate performance of the Clauss-CWA method with these target diseases. The Clauss-CWA method might be expected to provide additional opportunities to detect “hidden” acquired dysfibrinogenemia in the future. Second, the Clauss-CWA method could not analyze when a change in light transmittance would not be detected, such as with severe defects in fibrin polymerization^26–29^. In patients carrying these pathogenic variants, additional immunological measurements are required. Third, Clauss-CWA provided inaccurate results when analyzing higher levels of plasma functional fibrinogen. Actually, the calibration curve for eAg ranged from 0.58 to 4.60 g/L (Supplementary Fig. S2). Particularly with functional fibrinogen > 4.5 g/L, further dilution of plasma samples would be needed to obtain a correct Ac/eAg ratio. Finally, this autoanalysis software has only been developed for the Sysmex CN-series at present, and adaptation to other autoanalyzers has not been investigated. The availability for other photometric or viscoelastic systems should be assessed in future studies.

In conclusion, we found that Clauss-CWA offers a novel approach to detecting qualitative fibrinogen abnormalities, and is now ready for use in routine laboratory testing. The proposed cutoff of 0.65 for Ac/eAg ratio offered excellent sensitivity and specificity. This method, made available simply by upgrading the software, is expected to see use in many laboratories and may help detect cryptic fibrinogen abnormalities. We hope that the method we have developed will contribute to all laboratories, including those that do not specialize in thrombosis and hemostasis.

## Methods

### Patient selection

This study was designed as a single-center study, approved by the Nagoya University Hospital Ethics Committee (identification number: 2010-1038). We applied an opt-out method to obtain informed consent for participation in this study, which was conducted in accordance with the ethical standards laid down in the 1964 Declaration of Helsinki and its later amendments, by using the poster available for every patients in our hospital. In- and out-patients receiving blood coagulation tests in Nagoya University Hospital were randomly included if they showed a plasma fibrinogen level of 0.30–6.50 g/L as determined by routine laboratory testing, and had a sufficient volume of plasma sample available for additional measurements. Patients previously diagnosed with CD (n = 29) were also enrolled as instances of qualitative fibrinogen deficiency.

### Cohorts

The study comprised two independent cohorts: a training cohort and a validation cohort. The training cohort included clinical samples collected from June 2020 to July 2020. We screened plasma samples by the Ac/Ag ratio following additional measurement of fibrinogen antigen levels, and were considered normal quality for ratios > 0.70^30^. After excluding extra samples from the same participants, the residual plasma samples were defined as the normal group. Plasma samples from 15 patients with CD were then shuffled into the training cohort. The validation cohort included plasma samples randomly collected from August 2020 to October 2020. After excluding extra samples from the same participants, residual samples were defined as the control group. Plasma samples from 14 patients with CD (no overlaps against the training cohort) were shuffled into the control group and used as a validation cohort.

Data on available variables of interest included patient characteristics (age, sex), results of coagulation (prothrombin time and activated thromboplastin time), and liver function tests (alanine aminotransferase, aspartate aminotransferase, and total bilirubin).

### Sample collection

Blood was collected in polypropylene tubes with 0.109 mol/L of sodium citrate, and citrated plasma was prepared within 4 h after collection, as previously described^10^. Plasma samples were used for routine testing immediately after plasma separation, and residual samples were stored at – 80 °C until further fibrinogen analysis. Stored samples were thawed in a 37 °C water bath for 5 min and gently mixed well just before measurement.

### Autoanalysis software development

The autoanalysis software for qualitative analysis of plasma fibrinogen was developed and installed on a CN-6000 autoanalyzer (Sysmex, Kobe, Japan). The original method we previously established^10^ required CWA when calibrating the fibrinogen Ac, and the Min1 value was subsequently obtained and used to generate a calibration curve of eAg by manual calculation using a spreadsheet such as Excel^®^ (Microsoft, Redmond, WA, USA). The estimated specific activity (Ac/eAg ratio) was also calculated in the spreadsheet. The developed automatic analysis software enabled storage of multiple calibration curves for the different variables in the same assay, such as fibrinogen Ac and Min1. The representative calibration curve for eAg is shown in Supplementary Fig. S2. Actually, the calibration curve for fibrinogen Ac was generated, and simultaneously the calibration curve for eAg was created automatically. After validating the generated calibration curves of Ac and eAg, we could automatically and simultaneously obtain Ac, eAg, and Ac/eAg ratio from a single Clauss fibrinogen assay. Analytical flow was compared with the original method^10^ and the gold standard method (Clauss fibrinogen and immunological assay) in Supplementary Fig. S3.

### Fibrinogen assays

The Clauss fibrinogen assay was carried out using Thrombocheck Fib(L) reagent (TCFibL; Sysmex) and Dade^®^ Thrombin Reagent (Dade; Siemens Healthineer, Marburg, Germany) in the CN-6000 autoanalyzer. In this study, the measured value from the Clauss fibrinogen assay, indicating the amount of functional fibrinogen, was termed fibrinogen activity (Ac). Fibrinogen antigen (Ag) was measured using FactorAuto Fibrinogen (Q-may Corporation, Oita, Japan) according to a previous study^10^. Calibration curves were generated using Standard Human Plasma (Siemens Healthineer) for all fibrinogen assays.

Fibrinogen Ac, eAg, and Ag were measured at the same time for each plasma sample. In Clauss fibrinogen assays, plasma samples were diluted 2- or fourfold when fibrinogen Ac exceeded 4.50 g/L. In fibrinogen Ag measurements, plasma samples were diluted twice when Ag exceeded 5.00 g/L.

### Analytical precision

Quality control plasma N (CPN) and P (CPP) (Siemens, for Ac, and eAg) or control plasma N and L (Q-may, for Ag) were used to assess analytical precision of fibrinogen Ac, eAg, and Ag. Five repetitions of each measurement were performed for 6 days independently and the coefficient of variation (CV) was calculated.

### Statistical analysis

Prism 9 (GraphPad Software, San Diego, CA, USA) and MedCalc (MedCalc Software, Ostend, Belgium) was used for statistical analysis. Continuous variables are presented as median with range, and categorical variables are presented as percentiles. Continuous data between the two cohorts were compared with Mann–Whitney *U* tests, and categorical data used Fisher’s exact test.

In the training cohort, predictive values of Ac/Ag ratio and Ac/eAg ratio for CD from controls were assessed by receiver operating characteristic (ROC) curve analysis. Area under the ROC curve (AUROC) and corresponding 95% confidence intervals (CIs) and cutoff values for predicting CD were recorded. Optimal cutoff values were estimated using the Youden index^31^.

In the validation cohort, the predictive accuracy of cutoff values for Ac/Ag ratio and Ac/eAg ratio was assessed by sensitivity and specificity with corresponding 95% CIs and likelihood ratio (LR). Classification performance was also validated by precision–recall (PR) curve analysis. For all analyses, statistical significance was indicated by two-sided values of *P* < 0.001.

## Supplementary Information

Supplementary Figures.

## References

[1] Roberts HR, Stinchcombe TE, Gabriel DA (2001). The dysfibrinogenaemias. Br. J. Haematol..

[2] Casini A (2018). Diagnosis and classification of congenital fibrinogen disorders: communication from the SSC of the ISTH. J. Thromb. Haemost..

[3] Casini A (2015). Natural history of patients with congenital dysfibrinogenemia. Blood.

[4] Haverkate, F. & Samama, M. Familial dysfibrinogenemia and thrombophilia. Report on a study of the SSC Subcommittee on Fibrinogen. *Thromb. Haemost.* **73**, 151–161 (1995). 7740487

[5] Shapiro SE (2013). Clinical phenotype, laboratory features and genotype of 35 patients with heritable dysfibrinogenaemia. Br. J. Haematol..

[6] Simurda T (2020). Comparison of clinical phenotype with genetic and laboratory results in 31 patients with congenital dysfibrinogenemia in northern Slovakia. Int. J. Hematol..

[7] Wypasek E (2019). Genetic and clinical characterization of congenital fibrinogen disorders in Polish patients: Identification of three novel fibrinogen gamma chain mutations. Thromb. Res..

[8] Smith N (2018). Identification and characterization of novel mutations implicated in congenital fibrinogen disorders. Res. Pract. Thromb. Haemost..

[9] de Moerloose P, Casini A, Neerman-Arbez M (2013). Congenital fibrinogen disorders: an update. Semin. Thromb. Hemost..

[10] Suzuki A (2019). Clot waveform analysis in Clauss fibrinogen assay contributes to classification of fibrinogen disorders. Thromb. Res..

[11] Shima M (2013). Towards standardization of clot waveform analysis and recommendations for its clinical applications. J. Thromb. Haemost..

[12] Casini A (2020). From routine to research laboratory: Strategies for the diagnosis of congenital fibrinogen disorders. Hamostaseologie.

[13] Ozenne B, Subtil F, Maucort-Boulch D (2015). The precision–recall curve overcame the optimism of the receiver operating characteristic curve in rare diseases. J. Clin. Epidemiol..

[14] Saito T, Rehmsmeier M (2015). The precision–recall plot is more informative than the ROC plot when evaluating binary classifiers on imbalanced datasets. PLoS ONE.

[15] Miesbach W, Schenk J, Alesci S, Lindhoff-Last E (2010). Comparison of the fibrinogen Clauss assay and the fibrinogen PT derived method in patients with dysfibrinogenemia. Thromb Res.

[16] Skornova, I. *et al.* Use of fibrinogen determination methods in differential diagnosis of hypofibrinogenemia and dysfibrinogenemia. *Clin. Lab.*10.7754/Clin.Lab.2020.200820 (2021). 10.7754/Clin.Lab.2020.200820 33865248

[17] Sato, H. & Nakajima, A. Kinetic study on the initial stage of the fibrinogen-fibrin conversion by thrombin. (II) Application of enzyme kinetics to turbidimetrical method. *Thromb Res* **35**, 133–139. 10.1016/0049-3848(84)90208-1 (1984). 10.1016/0049-3848(84)90208-1 6474414

[18] Peyvandi F (2012). Epidemiology and treatment of congenital fibrinogen deficiency. Thromb. Res..

[19] Francis JL, Armstrong DJ (1982). Acquired dysfibrinogenaemia in liver disease. J. Clin. Pathol..

[20] Siddiq, N., Bergstrom, C., Anderson, L. D., Jr. & Nagalla, S. Bleeding due to acquired dysfibrinogenemia as the initial presentation of multiple myeloma. *BMJ Case Rep*. 10.1136/bcr-2019-229312 (2019). 10.1136/bcr-2019-229312 PMC6663320 31320370

[21] Arai S (2020). Acquired dysfibrinogenemia: Monoclonal lambda-type IgA binding to fibrinogen caused lower functional plasma fibrinogen level and abnormal clot formation. Int. J. Hematol..

[22] Kotlin R (2008). Acquired dysfibrinogenemia secondary to multiple myeloma. Acta Haematol..

[23] Inano S (2021). Acquired hypofibrinogenemia in a patient with multiple myeloma. Int. J. Hematol..

[24] Bang, N. U., Fletcher, A. P., Alkjaersig, N. & Sherry, S. Pathogenesis of the coagulation defect developing during pathological plasma proteolytic (“fibrinolytic”) states. III. Demonstration of abnormal clot structure by electron microscopy. *J. Clin. Invest.* **41**, 935–948. 10.1172/JCI104548 (1962). 10.1172/JCI104548 PMC290993 13864646

[25] Mischke R, Wolling H (2000). Influence of fibrinogen degradation products on thrombin time, activated partial thromboplastin time and prothrombin time of canine plasma. Haemostasis.

[26] Koopman J, Haverkate F, Briët E, Lord ST (1991). A congenitally abnormal fibrinogen (Vlissingen) with a 6-base deletion in the gamma-chain gene, causing defective calcium binding and impaired fibrin polymerization. J. Biol. Chem..

[27] Steinmann C (1994). A new substitution, gamma 358 Ser–>Cys, in fibrinogen Milano VII causes defective fibrin polymerization. Blood.

[28] Lounes KC (1999). Fibrinogen Bastia (gamma 318 Asp–>Tyr) a novel abnormal fibrinogen characterized by defective fibrin polymerization. Thromb. Haemost..

[29] Okumura N (1996). Fibrinogen Matsumoto I: a gamma 364 Asp–>His (GAT–>CAT) substitution associated with defective fibrin polymerization. Thromb. Haemost..

[30] Krammer B, Anders O, Nagel HR, Burstein C, Steiner M (1994). Screening of dysfibrinogenaemia using the fibrinogen function versus antigen concentration ratio. Thromb. Res..

[31] Youden WJ (1950). Index for rating diagnostic tests. Cancer.

