# Supplementary material for: Development and validation of a novel qualitative test for plasma fibrinogen utilizing clot waveform analysis
Source: Sci Rep. 2022 Jan 21;12:434. doi: 10.1038/s41598-021-04464-5 (PMC8782860; doi:10.1038/s41598-021-04464-5)
Supplement: Supplementary file 1 — Supplementary Figures. [file 41598_2021_4464_MOESM1_ESM.pdf]

Supplementary Information

Development and validation of a novel qualitative test for plasma fibrinogen utilizing clot waveform analysis

Atsuo Suzuki[1], Nobuaki Suzuki[2], Takeshi Kanematsu[3], Sho Shinohara[4], Hiroshi Kurono[4], Nobuo Arai[4], Shuichi Okamoto[3], Naruko Suzuki[5], Shogo Tamura[6], Ryosuke Kikuchi[1], Akira Katsumi[7], Tetsuhito Kojima[6,8], and Tadashi Matsushita[2,4]

[1] Department of Medical Technique, Nagoya University Hospital, Nagoya, Aichi, Japan
[2] Department of Transfusion Medicine, Nagoya University Hospital, Nagoya, Aichi, Japan
[3] Department of Clinical Laboratory, Nagoya University Hospital, Nagoya, Aichi, Japan
[4] Sysmex Corporation, Kobe, Hyogo, Japan
[5] Department of Hematology-Oncology, Nagoya University Graduate School of Medicine, Nagoya, Aichi, Japan
[6] Division of Cellular and Genetic Sciences, Department of Integrated Health Sciences, Nagoya University Graduate School of Medicine, Nagoya, Aichi, Japan
[7] Department of Hematology, National Centre for Geriatrics and Gerontology, Obu, Aichi, Japan
[8] Aichi Health Promotion Foundation, Nagoya, Aichi, Japan

Table S1. Precision of fibrinogen assays

		N	L
Thrombocheck Fib(L)	Ac	2.6	3.9
	eAg	2.1	4.7
	Ac/eAg	1.6	5.3
Dade Thrombin Reagent	Ac	2.6	4.0
	eAg	2.5	2.6
	Ac/eAg	2.2	3.7
FactorAuto Fibrinogen	Ag	3.1	3.7

Precision is represented as the coefficient of variation (%).
N, normal level; L, low level.

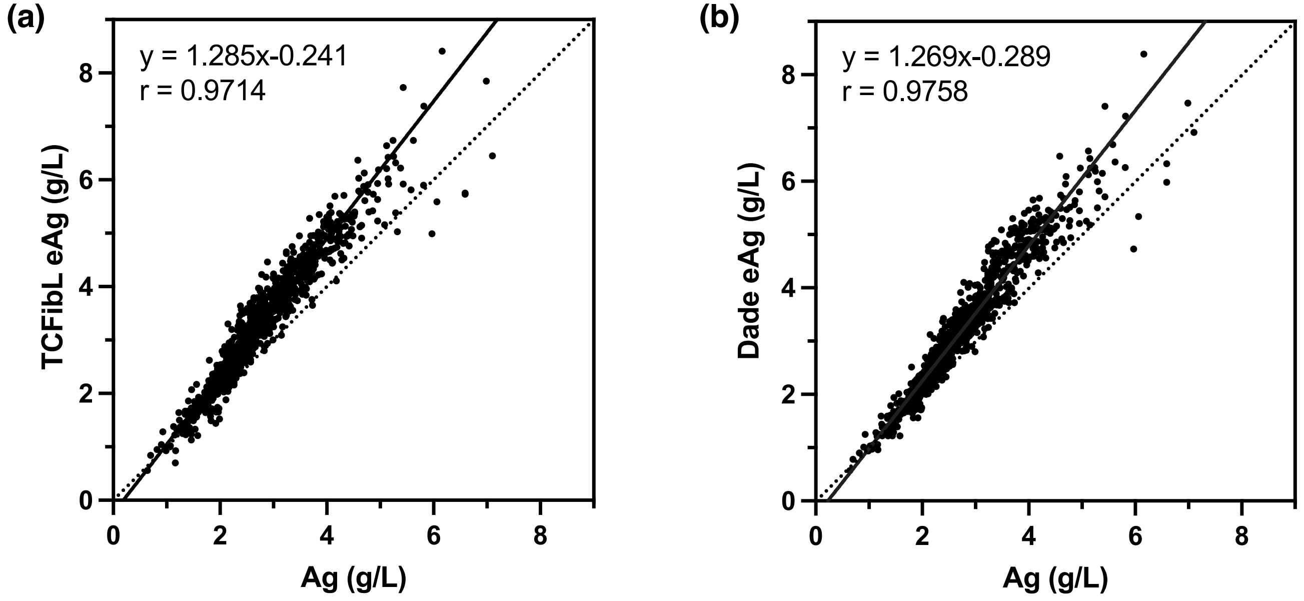

Supplementary Figure S1. Correlations between eAg and Ag

Correlations between Ag and eAg were analyzed in the whole cohort after excluding samples from patients with congenital (hypo)dysfibrinogenemia (n=1,013). The concentration of eAg was determined using two reagents, TCFibL (a) or Dade (b), then compared with Ag measured immunologically. Dotted lines indicate the y=x line. Solid lines represent linear regressions. Ag, fibrinogen antigen; eAg, estimated fibrinogen antigen; TCFibL, Thrombocheck FibL reagent; Dade, Dade thrombin reagent.

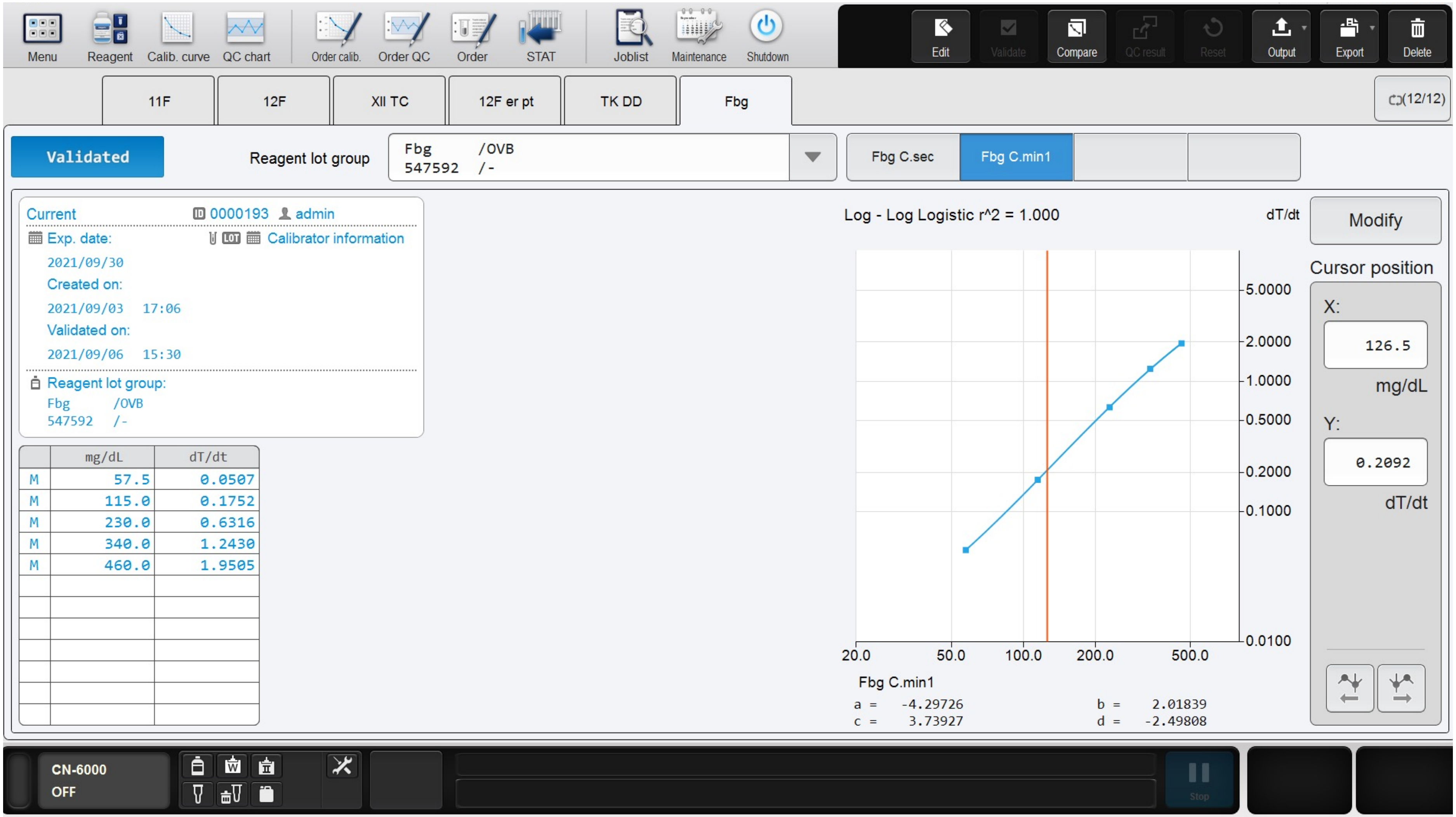

Supplementary Figure S2. An image of the calibration curve for eAg automatically generated in the Sysmex CN-6000.

This screen capture represents a calibration curve for eAg generated simultaneously with fibrinogen Ac calibration. The Sysmex CN-6000 software can store two calibration curves for different variables in the same assay. The image gives an example from software under development.

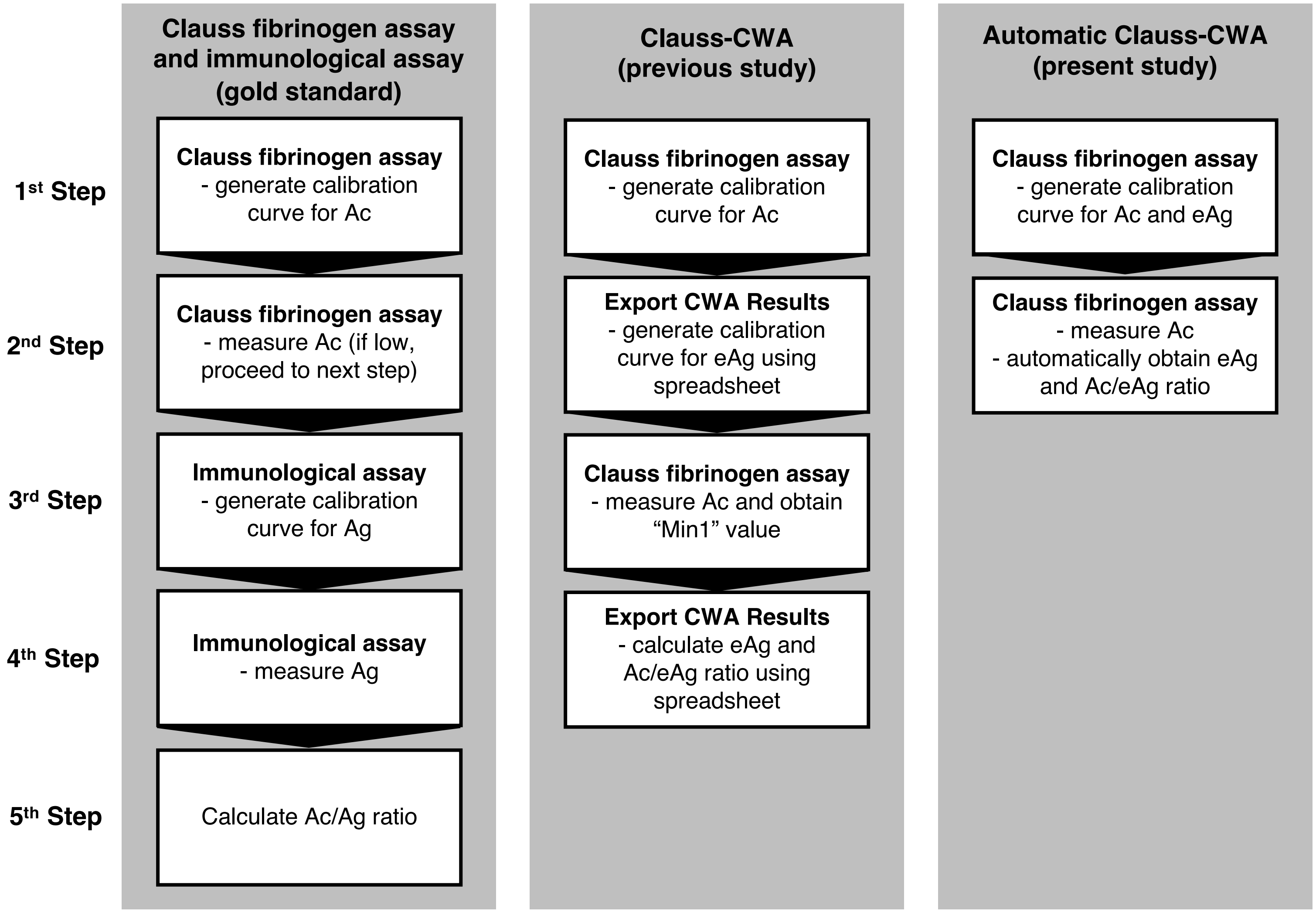

Supplementary Figure S3. Comparison of flowcharts for analysis of plasma fibrinogen in the three assays.

Analytical steps are compared among the three methods. The combination of Clauss fibrinogen and immunological assays is shown as the gold standard method in the left panel. The Clauss-CWA method was developed to allow automatic determination in the present study. CWA, clot waveform analysis; Ac, functional fibrinogen; Ag, fibrinogen antigen.
